# Mapping projects for expanding rapid HIV testing in key populations, Brazil, 2004-2021

**DOI:** 10.1590/0102-311XEN182323

**Published:** 2024-05-17

**Authors:** Lidiane da Silveira Gouvea Toledo, Ana Isabella Sousa Almeida, Francisco Inácio Bastos

**Affiliations:** 1 Conselho Nacional de Desenvolvimento Científico e Tecnológico, Brasília, Brasil.; 2 Escola Nacional de Saúde Pública Sergio Arouca, Fundação Oswaldo Cruz, Rio de Janeiro, Brasil.; 3 Instituto de Comunicação e Informação Científica e Tecnológica em Saúde, Fundação Oswaldo Cruz, Rio de Janeiro, Brasil.

**Keywords:** AIDS, HIV Testing, Nongovernmental Organizations, Health Governance, AIDS, Teste de HIV, Organizações Não Governamentais, Governança em Saúde, SIDA, Prueba de VIH, Organizaciones No Gubernamentales, Gobernanza

## Abstract

The HIV/AIDS epidemic remains a persistent and real issue, especially in key populations such as men who have sex with men (MSM), *travestis* and transgender persons. Projects for expanding rapid HIV testing are strategic initiatives aimed at the earliest possible identification of individuals’ serological status and thus early treatment, screening of sex partners, and upscaling of preventive actions to interrupt the transmission chain. This study thus maps, describes, and systematizes the projects for expanding rapid HIV testing implemented from 2004 to 2021 in Brazil, highlighting the on-going contribution of civil society organizations and discussing the interoperability and cooperation resulting from public governance processes. We selected 67 documents for analysis, including 30 scientific publications retrieved from electronic databases and 37 documents produced by government institutions and nongovernmental organizations (NGOs). *Find Out* (*Fique Sabendo*), *I Want to Get Tested* (*Quero Fazer*), *The Time is Now* (*A Hora É Agora*), *Live Better Knowing* (*Viva Melhor Sabendo*), and *Live Better Knowing Young* (*Viva Melhor Sabendo Jovem*) were the projects mapped. Results show that the projects have used strategies adapted to the key population, such as mobile testing units, peer education, and innovative community engagement approaches. Such actions were enabled by effective cooperation and interoperability between participating stakeholders, especially NGOs.

## Resumo

A epidemia de HIV/aids está longe de terminar. Ainda é muito real, especialmente em populações-chave, como homens que fazem sexo com homens (HSH), travestis e pessoas transgênero. Projetos de ampliação da testagem rápida anti-HIV são iniciativas estratégicas que visam à identificação mais precoce possível do *status* sorológico dos indivíduos e, consequentemente, ao tratamento precoce, à triagem de parceiros sexuais e à ampliação das ações preventivas para interrupção da cadeia de transmissão. Assim, este estudo se propõe a mapear, descrever e sistematizar os projetos de expansão da testagem rápida para HIV realizados de 2004 a 2021 no Brasil, destacando a contribuição em curso das organizações da sociedade civil e discutindo a interoperabilidade e a cooperação resultantes dos processos de governança pública. Foram selecionados 67 documentos para análise, incluindo 30 publicações científicas recuperadas de bases de dados eletrônicas e 37 documentos produzidos por instituições governamentais e organizações não governamentais (ONGs). Os projetos mapeados foram: *Fique Sabendo*, *Quero Fazer*, *A Hora É Agora*, *Viva Melhor Sabendo* e *Viva Melhor Sabendo Jovem*. Os resultados mostram que os projetos utilizaram estratégias adaptadas à população-chave, como unidades móveis de testagem, educação entre pares e abordagens inovadoras de engajamento comunitário. Tais ações foram possíveis graças à cooperação e interoperabilidade efetivas entre as partes interessadas participantes, especialmente as ONGs.

## Resumen

La epidemia de VIH/SIDS está lejos de terminar. Sigue siendo muy real, sobre todo en poblaciones clave, como hombres que tienen sexo con hombres (HSH), travestís y personas transgénero. Proyectos para ampliar las pruebas rápidas anti-VIH son iniciativas estratégicas que tienen el objetivo de identificar el estado serológico de las personas lo antes posible y, consiguientemente, hacer el tratamiento precoz, el triaje de las parejas sexuales y ampliar las acciones preventivas para interrumpir la cadena de transmisión. Así, este estudio tiene el objetivo de mapear, describir y sistematizar los proyectos de expansión de las pruebas rápidas del VIH realizados entre 2004 y 2021 en Brasil, resaltando la contribución en curso de los organismos de la sociedad civil y discutiendo la interoperabilidad y la cooperación que resultan de los procesos de gobernanza pública. Se seleccionaron 67 documentos para el análisis, entre ellos 30 publicaciones científicas recuperadas de bases de datos electrónicas y 37 documentos producidos por instituciones gubernamentales y organizaciones no gubernamentales (ONGs). Los proyectos mapeados fueron: *Para que Sepas* (*Fique Sabendo*), *Quiero Hacer* (*Quero Fazer*), *La Hora Es Ahora* (*A Hora É Agora*), *Viva Mejor Sabiendo* (*Viva Melhor Sabendo*) y *Viva Mejor Sabiendo Joven* (*Viva Melhor Sabendo Jovem*). Los resultados demuestran que los proyectos utilizaron estrategias adaptadas a la población clave, como las unidades móviles de prueba, educación entre pares y enfoques innovadores de participación comunitaria. Estas acciones fueron posibles gracias a la cooperación e interoperabilidad efectivas entre las partes interesadas participantes, sobre todo las ONGs.

## Introduction

In Brazil, counseling and testing for identifying HIV are available nationwide in the Brazilian Unified National Health System (SUS, acronym in Portuguese) health networks and in community-based civil society organizations ^1^ ^,^ ^2^. However, according to data from the Brazilian Ministry of Health on positive test results in 2020, 20% of adults aged 25 to 29 years, 34% of those aged 30 to 49 years, and 45% of those over 50 years old received a late HIV diagnosis. In other words, they failed to benefit from this testing structure and only requested care after presenting clinical symptoms ^3^. These data are worrisome, since early detection of any health condition, whether communicable or not, allows higher remission odds and even cure, which become more complex once the condition is already installed, especially in advanced stages ^4^.

Access to HIV testing can be greatly limited in key populations such as men who have sex with men (MSM), *travestis* and transgender women due to structural barriers to access such as unstable or unfavorable living and housing conditions, lack of flexible office hours, stigma and prejudice within health services, and environments with little or no sensitivity to gender issues and sexual diversity ^5^ ^,^ ^6^.

In this scenario, rapid testing projects targeting key populations have become the focus of Brazilian Minsitry of Health, since testing is a critical point and portal of entry for the continuing care of HIV/AIDS. Examples of such projects are *Find Out* (*Fique Sabendo*), *I Want to Get Tested* (*Quero Fazer*), *The Time is Now* (*A Hora É Agora*), *Live Better Knowing* (*Viva Melhor Sabendo*), and *Live Better Knowing Young* (*Viva Melhor Sabendo Jovem*) ^7^ ^,^ ^8^.

Technical and narrative progress reports produced by these projects however are usually seen only by the involved funding and implementing agencies, hindering the publicization and critical evaluation of such projects that would otherwise help guide public policies in health care. Few people have access to these reports, which requires a painstaking search since they are usually not indexed in standard scientific databases and sometimes are not even available for download on the internet. To a great extent, they represent a kind of “gray literature” (originally a set of information defined as “classified”, stored in gray folders). Curiously, we were unable to locate more comprehensive reviews of gray literature precisely in the form of unindexed publications ^9^.

Thus, this study mapped, systematized, and described the main projects aimed at expanding rapid HIV testing focused on MSM, *travestis* and transgender women implemented from 2004 to 2021 in Brazil, emphasizing the role of civil society in its interrelations with the government.

## Methodology

An exploratory study was conducted based on a documentary analysis of technical-scientific reports and search of indexed and unindexed literature on projects aimed at expanding rapid HIV testing in key populations, namely: *Find Out*, *I Want to Get Tested*, *The Time is Now*, *Live Better Knowing*, and *Live Better Knowing Young* - hereinafter the projects will be mentioned by their English translation. Technical-scientific reports were obtained by contacting (1) the Department of Chronic Conditions and Sexually Transmitted Infections (DCCI), Brazilian Ministry of Health; (2) technical areas on sexually transmitted infections (STI) and AIDS of the 26 states and Federal District health departments; and (3) 134 nongovernmental organizations (NGOs) identified by means of the repository of information on civil society organizations working with HIV/AIDS, available on the DCCI website (https://www.gov.br/aids/pt-br), the scope of which includes rapid HIV testing and/or serving the key population.

We made four contact attempts via three different communication channels on alternating days, weeks, and hours, listed in order of priority: e-mails, telephone calls, and an instant message app from July 2021 to January 2022. Having made contact, we asked the following questions: (1) Have you conducted any actions/activities/campaigns/cooperative projects and/or research aimed at expanding HIV testing for *travestis*, transgender women and/or the MSM population from 2004 to 2021? (2) If yes, are there technical reports, executive summaries, communications, articles, theses, and dissertations that report results of such actions (e.g.: target public reached, number of transgender women, *travestis* and/or MSM tested, number of transgender women, *travestis* and/or MSM with positive test results), with open access and that can be sent to our research team?, and (3) Is the technical area aware of any municipality or state NGO that has explicitly promoted rapid HIV testing expansion in the aforementioned populations? This last question was only addressed to the states and Federal District’s technical areas.

We included technical-scientific reports that cited any activity or project aimed at expanding rapid HIV testing in the key population between 2004 and 2021, considering testing activities or projects geared towards the target public when the activity or project was not part of the original protocol. Exclusion criteria consisted in not discernibly citing MSM and/or *travestis* and transgender women, not citing the year in which the activity or project was conducted, and duplicate documents.

Literature search related to the *Find Out*, *Live Better Knowing*, *Live Better Knowing Young*, *The Time is Now* and *I Want to Get Tested* was performed in January 2022 in the MEDLINE/PubMed, LILACS/VHL, SciELO Brazil, Google Scholar, Catalogue of theses and dissertations of the Brazilian Coordination for the Improvent of Higher Education Personnel (CAPES Catalogue of Theses and Dissertations), Brazilian Open Access Portal of Publications and Scientific Data (OASIS/IBICT), Institutional Repository of the Oswaldo Cruz Foundation (ARCA/Fiocruz) databases using standardized search equations and complementary manual search strategies (e.g., searches in specific periodicals, websites, abstracts, contact with researchers, and reference lists). The following search strategy was used in all the databases and unindexed documents with the necessary adjustments: ((“The Time is Now”) OR (“Live Better Knowing”) OR (“Live Better Knowing Young”) OR (“Find Out”) OR (“I Want to Get Tested”)).

**Figure 1:**
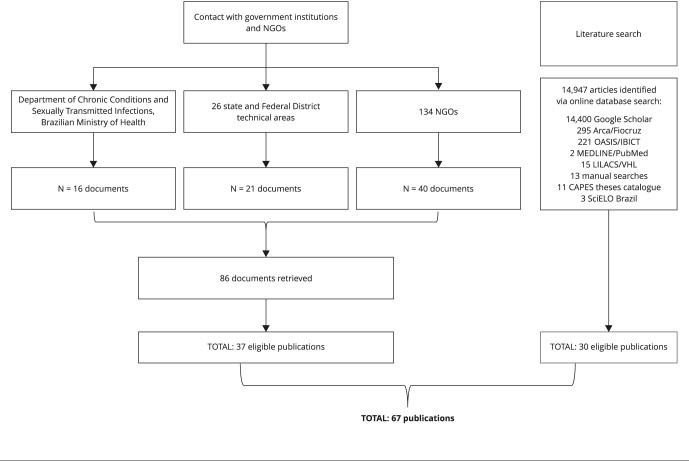
Search and selection of documents for analysis.

We later included studies published from 2004 to 2021 that involved the key populations and cited at least one of the selected projects. Duplicates and articles that did not afford full access were excluded. Figure 1 illustrates the article selection steps.

NGO: nongovernmental organization.

## Results

### Comprehensive perspective

Brazil’s Federal Government system provides for decentralized administration of the SUS ^10^, affording the states and municipalities the autonomy to define local health actions in keeping with the basic SUS principles. We thus expected to find projects for expanding rapid HIV testing in key populations led by states and municipalities, which proved a false assumption.

Brazilian state governments appear to focus on promoting regular rapid testing services at the primary health care level and testing and counseling centers for the general population. States also reported that few municipalities led such initiatives, corresponding to a small percentage of the more than 5,500 Brazilian municipalities.

In Brazil, projects for expanding HIV testing in key populations have been conducted mostly by civil society in direct partnership with Brazilian Ministry of Health, which characterizes its previous and current department structure via “calls for projects” (public bids). States, in turn, promote or fund actions to expand rapid testing among the lesbian, gay, bisexual, transgender, queer, intersex, asexual, pansexual, and non-binary (LGBTQIAPN+) population locally in partnership with NGOs, providing transportation and tests. Four of the six projects also received funding and technical cooperation from international and domestic agencies such as the U.S. Agency for International Development (USAID) (*I Want to Get Tested*); the United Nations Children’s Fund (UNICEF; *Find Out Young*); the Oswaldo Cruz Foundation (Fiocruz, acronym in Portuguese), scientific partner institution in charge of executing the *The Time is Now* project; and the National Conference of Bishops of Brazil (CNBB, acronym in Portuguese), by providing technical support.

### Specific projects

####  I Want to Get Tested project (2008-2014): networks and partnerships

Launched in 2008, the *I Want to Get Tested* project was funded by USAID and implemented by two Pact-Brazil (2008-2010) and the Space for Prevention and Humanized Care (EPAH) (2011-2014). It sought to expand HIV testing among *travestis* and MSM using flexible hours and mobile units (e.g., trailers) to expand access to HIV diagnosis, counseling and testing, besides promoting prevention and healthcare activities ^11^. São Paulo, Rio de Janeiro, Recife (Pernambuco State), Brasília, and Fortaleza (Ceará State) were the cities covered by the project. A total of 26,785 tests were performed, 8,879 of which among the gay, MSM and *travesti* population. Interestingly, in 2009-2010 fewer than 3% of all the participants tested in the five municipalities were *travesti*, showing low adherence by this population ^12^. This finding corroborates the study by Kulick ^13^, who reported that the population of *travestis* is marked by profound marginalization and complex interaction with various institutions, including health services.

The project team included at least two peer educators, one counselor, a technician to perform the finger-stick test, and a nurse, with the services network supported by state and municipal AIDS programs and consisting of primary care units, testing and counseling centers (TCCs), specialized services, and NGOs ^14^. The three-stage strategy involved (1) finger-stick rapid testing (i.e., peripheral blood) in the TCCs, (2) counseling and testing in the NGOs’ headquarters, and (3) testing in trailers that visited sites frequented by the LGBTQIAPN+ population in each municipality. The latter highlighted the importance of peer educators, since they were responsible for inviting users for testing through social awareness-raising actions.

Brasília and Recife established partnerships with LGBTQIAPN+ nightclubs and bars for parking the trailers near their premises. However, an user satisfaction evaluation ^15^ found that “fixing” the mobile service in one place exhausted the possibilities of performing new tests, since most frequenters had already been tested, besides posing a logical contradiction by breaking with the project’s dynamism. Fixed and mobile strategies should be independent but complementary, as reported in successful international experiences ^16^.

Changes in the adoption of safer behaviors and practices such as more frequent condom use, reduced geographic barriers with the use of roving trailers and actions in LGBTQIAPN+ socialization venues were positive points of the project. However, it faced difficulties in connecting municipal and state health departments to logistics services for implementing a mobile service (trailers). Box 1 summarizes the characteristics of the *I Want to Get Tested* project.

**Box 1:** Characteristics of the *I Want to Get Tested* project (2008-2014).

***I WANT TO TEST* (2008-2014)** **(10 DOCUMENTS ANALYZED)**						
PROJECT SCOPE		PROJECT GOVERNANCE		TOTAL OF TESTS PERFORMED	TOTAL OF TESTS IN TARGET POPULATIONS	POSITIVE RESULTS AMONG KEY POPULATIONS
Key population	*Travestis* and MSM	Funding	Provided by the U.S. Agency for International Development	(2008-2014) 26,785	(2008-2014) 8,879	1,032 people were diagnosed with HIV, but there is no information on positive results (seroreagents for HIV) stratified by key population
Objective(s)	Expand HIV testing among *travestis* and MSM in alternating hours using trailers to help reduce access barriers to prevention and healthcare in this population, thereby expanding access to early HIV diagnosis, and voluntary counseling and testing	Implementation	Program initially coordinated by Pact Brazil (2008-2010) and later by the Space for Prevention and Humanized Care organization. Expansion of rapid testing in TCCs was followed by implementation of voluntary counseling and testing in NGOs and later in trailers, with the participation of peer educators			
Services provided	Voluntary counseling and testing in two sites: (1) Fixed locations: nongovernmental organizations that serve the LGBT population and the TCC (2) Mobile units: trailers sattioned in areas frequented by LGBT public	Stakeholders	NGO leaders, administrators, healthcare workers, peer educators (members of the LGBT community), specialists			
		Participation of state and municipal networks	Provision of prevention supplies and human resources (public employees), peer training, and project publicization			
Intervention sites	São Paulo, Rio de Janeiro, Recife (Pernambuco State), Brasília, and Fortaleza (Ceará State)	Inter-sector network	Primary care units, TCC, specialized services, and NGOs			

LGBT: lesbian, gay, bisexual, and transgender; MSM: men who have sex with men; NGO: nongovernmental organization; TCC: testing and counseling center.

Source: prepared by the authors.

####  Find Out Young and Live Better Knowing Young projects (2013-2021): including the youth 

The pilot project *Find Out Young* was first implemented in Fortaleza and Porto Alegre (Rio Grande do Sul State) from 2013 to 2015, reaching more than 2,000 adolescents and young adults. In 2016 it was expanded to include the municipalities of São Paulo, Manaus (Amazonas State), Belém (Pará State), and Recife, changing its name to *Live Better Knowing Young* in response to the Department of HIV/AIDS, Tuberculosis, Viral Hepatitis, and Sexually Transmitted Infections (DDAHV), Brazilian Ministry of Health, new strategy for awareness-raising among the younger population regarding rapid HIV testing.

An initiative of the UNICEF, who funded and implemented the actions at the municipal level, the project included the Municipal Health Department, NGOs, and networks of adolescents and young adults as co-participant organizations. We found no documents that explicitly cited participation by the Brazilian Ministry of Health. Project implementation required a well-structured municipal healthcare network including combined prevention, testing, and patient uptake and treatment adherence. UNICEF produced documents ^17^ ^,^ ^18^ systematizing the necessary actions to be taken by municipalities to prepare to serve vulnerable populations, including (1) identification of hotspots (where the target public concentrated), (2) team training cycles, and (3) mobilization of adolescents and youth for sexual health promotion in schools and activities in youth detention centers. The project proposed to increase access to testing for HIV and other STIs and to encourage early treatment, as well as offering health education for young gays/MSM in youth detention centers and schools ^17^.

Testing, uptake, and treatment flow ^17^ was organized as follows: a trailer was set up at the hotspots and the youth mobilizers invited their peers to be tested. Testing could be via rapid diagnosis (finger-stick) performed by a healthcare worker or oral fluid collection by youth mobilizers. Individuals with negative test results received post-test counseling and were dismissed after receiving informational materials and prevention supplies (condoms and lubricant gel). Confirmed seropositive individuals were scheduled immediately for medical appointments at a primary care units, supported from medical appointment to treatment onset by a youth mobilizer.

Peer education significantly increased HIV testing and diagnoses and expanded the uptake and treatment adherence of adolescents and youth with HIV. Partnership with the “Rede Cuca” network encouraged its young frequenters to discover their serological status. However, since the UNICEF funding was interrupted in 2016 (as agreed in the initial project proposal), it is unclear in the available documentation whether the actions are still being funded by Brazilian Ministry of Health or led by the municipalities.

Only two of the six municipalities produced robust data on the number of tests performed in the key population. In Fortaleza, TCC was the most frequently accessed testing site, whereas in Porto Alegre it was the mobile unit ^18^ (Box 2). Only one document cites the project’s continuity (*Live Better Knowing Young*, Recife), but without information on funding. During the COVID-19 pandemic, the project began to offer weekly testing in a NGO headquarters and focused on distributing home self-tests, preceded by pre- and post-test counseling.

**Box 2:** Characteristics of the *Find Out Young*/*Live Better Knowing Young* project (2008-2014).

***FIND OUT YOUNG*/*LIVE BETTER KNOWING YOUNG* (2008-2014)** **(3 DOCUMENTS ANALYZED)**						
PROJECT SCOPE		PROJECT GOVERNANCE		TOTAL OF TESTS PERFORMED	TOTAL OF TESTS IN TARGET POPULATIONS	POSITIVE RESULTS AMONG KEY POPULATIONS
Key population	Gay and/or MSM adolescents and young adults	Funding	Provided by the United Nations Children’s Fund	Fortaleza (Ceará State)	Fortaleza (Ceará State)	Fortaleza (Ceará State)
Objective(s)	Increase access to voluntary testing for HIV and other STIs, expanding treatment adherence and conducting effective actions health prevention and promotion through socioeducational measures for gays and MSM in school settings	Implementation	Pilto project in two sites: Fortaleza (Ceará State) and Porto Alegre (Rio Grande do Sul State), and subsequent expansion to four more sites (Manaus - Amazonas State, Belém - Pará State, Recife - Pernambuco State, São Paulo). Implementation was preceded by training workshops for healthcare workers and youth mobilizers. After supply allocation and training, the activities were conducted in mobile units in socialization venues frequented by target public	(2014-2015) Mobile unit 1,208 tests TCCs N = 3,899	(2014-2015) 309 tests (homosexual, bisexual, MSM, *travestis*)	33 people were diagnosed with HIV, of which 32 identified themselves as homosexuals, travestis, MSM or bisexuals
Services provided	Service provided in obile units with direct prevention and risk minimization methods: rapid testing for HIV, syphilis, and viral hepatitis pereformed by a healthcare worker; rapid screening test (oral fluid sample taken by a youth mobilizer); and distribution of condoms and lubrificant gel	Stakeholders	Youth mobilizers, administrators, healthcare workers	Porto Alegre (Rio Grande do Sul State)	Porto Alegre (Rio Grande do Sul State)	Porto Alegre (Rio Grande do Sul State)
		Participation of state and municipal networks	Strategic support for testing, training adolescents and youth mobilizers, training of healthcare personnel	(2014-2015) Mobile units 1,362 tests	(2014-2015) 90 tests (MSM)	10 people diagnosed with HIV, of which 3 were MSM
Intervention sites	Ceará State, Rio Grande do Sul State, Amazonas State, Pará State, Pernambuco State, Pernambuco State, São Paulo State	Inter-sector network	Universities, NGOs, TCCs, psychosocial support group, schools, socioeducational centers	Recife (Pernambuco State)	Recife (Pernambuco State)	Recife (Pernambuco State)
				2021 215 self-tests distributed 85 tests at the headquarters	No information on category accessed	No information on HIV tests results

MSM: men who have sex with men; NGO: nongovernmental organization; STI: sexually transmitted infection; TCC: testing and counseling center.

Source: prepared by the authors.

####  Live Better Knowing project (2014-2022): testing innovation 

Inspired by the *I Want to Get Tested* project, the government launched the *Live Better Knowing* project in 2014 aiming to expand HIV testing among key populations based on a then innovative strategy: rapid HIV testing with oral fluid samples (systematic use of oral testing at international sites corresponds to this period or immediately afterwards). Unlike the *I Want to Get Tested* project, *Live Better Knowing* focused not only on MSM, gays, transgender persons, and *travestis*, but expanded its scope to include sex workers and people who use substances and was also the first (in conjunction with *Live Better Knowing Young*) in Brazil to collect oral samples (by individuals who did not necessarily have health training). In the previous project, the activists were peer educators, responsible for recruiting, raising awareness, and embracing the target public without however collecting biological samples.

*Live Better Knowing* is a project currently underway with multiple partnerships. It is funded by the Brazilian Ministry of Health via a letter of agreement with the United Nations Office on Drugs and Crime (UNODC) and is supported by the state and municipal health departments, with NGOs as the executive institutions. Project implementation involved selecting several NGOs known for leading activities with the key populations. The NGOs received a letter of invitation informing them of the project’s scope and were required to submit a formal proposal to receive funding. Of the 40 NGOs contacted, 34 were selected, and the *Live Better Knowing* project was conducted in 36 cities in all five of Brazil’s major geographic regions.

The project benefited from a mutual contribution: the Brazilian Ministry of Health was responsible for training 74 educators/NGO members, whereas the states and municipalities provided technical support within their territories. Educational materials and prevention supplies were provided by all government levels (federal, state, and municipal). NGOs were responsible for recruiting peer educators and conducting the testing activities with pre- and post-test counseling in sites frequented by the target public, such as LGBTQIAPN+ venues (e.g. bars, saunas, and clubs). The testing activities were well-received by the key population, but some participants were embarrassed to be tested in spaces for group socialization. International literature has cited the possible exposure of tested individuals, especially as members of stigmatized populations ^19^.

Although data is disaggregated by key population - unlike the *I Want to Get Tested* project -, the available results on the number of tests performed usually overlap in time, thus hindering an evaluation per year (Box 3).

**Box 3:** Characteristics of the *Live Better Knowing* project (2014-2022).

***LIVE BETTER KNOWING* (2014-2022)** **(24 DOCUMENTS ANALYZED)**						
PROJECT SCOPE		PROJECT GOVERNANCE		TOTAL OF TESTS PERFORMED	TOTAL OF TESTS IN TARGET POPULATIONS	POSITIVE RESULTS AMONG KEY POPULATIONS
Key population	Gays, MSM, *travestis*, transgender individuals, transsexual persons, sex workers, and substance users	Funding	Project funded by the Brazilian Ministry of Health by means of joint letter of agreement with the U.N. Office on Drugs and Crime, based on project document (PRODOC BRAK57)	(2014-2018) 173,929 (2018-2019) 45,821 (2021-2022) 56,433	(2014-2015) 1,125 *travestis*, 488 transgender persons, 5,364 gays and MSM (2018-2019) 1,077 *travestis*, 1,114 transgender persons, 9,049 gays and MSM (2021-2022) 564 *travestis*, 1,185 transgender persons, 9,593 gays and MSM	(2014-2015) 2,783 people diagnosed with HIV, of which 425 were gays and MSM and 629 transgender women and *travestis* (2018-2019) 626 people diagnosed with HIV, of which 263 were gays and MSM and 34 transgender women and 33 *travestis* (2021-2022) 733 people diagnosed with HIV, of which 258 were gays and MSM and 67 transgender women and 26 *travestis*
Objective(s)	Expand voluntary and timely HIV testing for persons in contexts of vulnerability	Implementation	Letter of invitation to NGOs selected according to leadership in activities for the target populations. NGOs had to present a formal proposal for project execution. Of the 40 organizations invited, 34 submitted the required documents. After approval, a contract was signed to provide funding, prevention supplies, and training in partnership with the respective municipal and state health departments			
Services provided	Provision of rapid tests with oral fluid samples in socialization spaces	Stakeholders	Partner NGO members, administrators, and peer educators			
		Participation of state and municipal networks	Both provided technical support in their respective areas, besides prevention supplies, educational materials, and training needed			
Intervention sites	Acre State, Amazonas State, Bahia State, Ceará State, Federal District, Espírito Santo State, Goiás State, Manaus State, Minas Gerais State, Pará State, Piauí State, Pernambuco State, Paraíba State, Roraima State, Rio Grande do Norte State, Santa Catarina State, São Paulo State, Sergipe State, Mato Grosso do Sul State	Inter-sector network	Primary care units, referral services, U.N. Office on Drugs and Crime, Coordination of Prevention and Social Networking, Coordination of Management and Governance, Coordination of Laboratories, Advisory Division for Monitoring and Evaluation			

MSM: men who have sex with men; NGO: nongovernmental organization.

Source: prepared by the authors.

Despite prioritizing key populations, many individuals tested by the *Live Better Knowing* project belonged to other several impoverished, underserved strata. In 2018-2019, 45 participating NGOs performed 45,660 tests. Interestingly, 21,903 (47.9%) of the people tested were cisgender women, of whom ~33% were sex workers.

Finally, some NGOs reported difficulty in conducting the activities due to a disconnect between the state and municipal programs, which failed to understand their respective roles in the project. Other important factors that impacted project development were the delays in federal transfers and the COVID-19 pandemic, pointed out as the main cause for the low number of tests in 2020-2022 given the impossibility of performing activities outside clinic walls.

####  The Time is Now project (2014-2022): community engagement 

Launched in 2014 in Curitiba city, Paraná State, the project aimed to expand detection of HIV infection among gays and other MSM and to encourage their uptake by health services for treatment. Funded by the U.S. President’s Emergency Plan for AIDS Relief (PEPFAR), the project was implemented by means of a cooperative agreement between the Sergio Arouca National School of Public Health, Oswaldo Cuz Foundation (ENSP/Fiocruz, acronym in Portuguese) and the U.S. Centers for Disease Control and Prevention (CDC).

Other institutional partners included the DDAHV, the Curitiba Municipal Health Department, the Federal University of Paraná (UFPR, acronym in Portuguese), Grupo Dignidade (a Curitiba-based NGO), the Evandro Chagas National Institute of Infectious Diseases (INI/Fiocruz, acronym in Portuguese), and local stakeholders such as health system administrators, researchers, activists, healthcare workers, and members of the LGBTQIAPN+ community, as well as a robust inter-sector network consisting of primary care units, HIV/AIDS referral centers, street outreach clinics, and social services. States and municipalities were responsible for distributing prevention supplies, logistics, and project publicization. 

Its first phase (2014-2017) was conducted in Curitiba by offering rapid finger-stick tests in trailers stationed at strategic locations, an LGBTQIAPN+ NGO, a TCC, and street outreach clinics ^2^. Oral fluid test (self-test) could be picked up at the post office or Brazilian Ministry of Health popular pharmacies following registration on the project’s website. Excepting the street outreach clinic, which had its own staff, the other sites recruited personnel for the following jobs: peer educators, sample collectors, counselors, and engagers (called “linkers”). The innovative strategy for increasing community engagement was performed as follows: peer educators approached the target public, who discovered the testing venue via campaign on social networks and media. After testing, in case of a seropositive result, the uptake - “linkage” - began, in which a healthcare worker supported and registered the individual in the city’s referral health services to initiate treatment. From 2014 to 2017, 1,750 MSM underwent HIV testing for the first time, and 90% of seropositive results were linked to the HIV/AIDS services ^20^. *The Time is Now* project reached more than 23,000 gays/MSM by peer approach, and the digital platform provided more than 6,000 test kits ^20^ ^,^ ^21^ (Box 4).

**Box 4:** Characteristics of *The Time is Now* project (2014-2022).

***THE TIME IS NOW* (2014-2022)** **(30 DOCUMENTS ANALYZED)**						
PROJECT SCOPE		PROJECT GOVERNANCE		TOTAL OF TESTS PERFORMED	TOTAL OF TESTS IN TARGET POPULATIONS	POSITIVE RESULTS AMONG KEY POPULATIONS
Key population	MSM	Funding	Grant from the Global AIDS Program, U.S. Centers for Disease Control and Prevention	(2015-2017) 7,040 (pilot study in Curitiba, Paraná State) (2020-2021) 7,866 self-tests - Curitiba (Paraná State), Campo Grande (Mato Grosso do Sul State) and Florianópolis (Santa Catarina State)	(2015-2017) 2,994 tests in MSM	(2015-2017) No information on the total number of people diagnosed with HIV, but it was reported that 256 MSM tested positive during the period under analysis
Objective(s)	Expand HIV infection in gay men and MSM, with referral to health services for treatment following positive test results	Implementation	Cooperative agreement with Fiotec, with support from the ENSP and INI, both managed by Fiocruz, Brazilian Ministry of Health, Municipal Health Department, UFPR, NGO Dignidade. Pilot project in Curtiba (Paraná State) and subsequent expansion			
Services provided	Couseling and testing: fixed sites (NGO Dignidade, Center for Orientation and Counseling), mobile sites (trailers managed by the Municipal Health Department, and street outreach clinics); self-test via internet (E-testing); community engagement service (optional support from linker and referral to health service)	Stakeholders	Administrators, activists, healthcare workers, ad members of the LGBTQIAPN+ community			
		Participation of state and municipal networks	Both supported and assisted with distribution of prevention supplies, logistics, and project publicization			
Intervention sites	Curitiba (Paraná State), Campo Grande (Mato Grosso do Sul State), Florianópolis (Santa Catarina State), Fortaleza (Ceará State) , Porto Alegre (Rio Grande do Sul State)	Inter-sector network	Logistics service, social services, primary health units and referral centers

ENSP: Sergio Arouca National School of Public Health; Fiocruz: Oswaldo Cruz Foundation; Fiotec: Fiocruz Support Foundation; INI: Evandro Chagas National Institute of Infectious Diseases; MSM: men who have sex with men; NGO: nongovernmental organization; UFPR: Federal University of Paraná.

Source: prepared by the authors.

Project expansion and improvement occurred in the second phase (2018-2022) upon launch of the eCOA clinic (Clinical Outcome Assessment), geared exclusively towards detection, prevention, and timely treatment of HIV and other STIs in the target population. In this phase, Campo Grande (Mato Grosso do Sul State), Florianópolis (Santa Catarina State), Porto Alegre, and Fortaleza also joined the project, which had the following goals: prevention and diagnosis via regular testing for HIV and other STIs, pre-exposure prophylaxis (PrEP), post-exposure prophylaxis (PEP), partner notification (index case testing), immediate treatment of HIV, STIs, and opportunistic infections with support from the “linkage” team, and active search of patients in case of treatment dropout ^2^.

Approaching group socialization sites proved to be an effective strategy, but incurred two difficulties: (1) understanding that the project was aimed at only gay men and MSM and (2) fear that the testing site would be identified as an exclusively LGBTQIAPN+ venue, thus associating it inadvertently and prejudicially to HIV infection. But these did not ultimately pose a barrier to project performance.

Finally, access expansion and the equitable implementation of prevention, diagnosis, care, and engagement of MSM in healthcare services were only possible due to the collaborative and integrative model involving various government institutions and nongovernmental organizations, which reduced the existing barriers to continuity of care by means of proactive engagement, one of the project’s main strengths ^2^ ^,^ ^21^.

## Discussion

The activities proposed by the projects for expanding HIV testing in MSM, *travestis* and transgender women in Brazil benefited from wide collaboration between social actors, government institutions, and nongovernmental organizations, besides international agencies.

### Recruitment strategies, rapid HIV testing, and continuity of care

Expanding rapid HIV testing in MSM, prison inmates, people who use substances with conditions associated with harm and addiction, sex workers, transgender persons, and adolescents and youth from “key populations” has been a global priority, as the agendas and technological innovations in patient care and prevention prioritize populations with higher HIV prevalence ^22^. Even though the original goal by the World Health Organization (WHO) to “*end AIDS by 2030*” ^23^ is currently understood by WHO experts as infeasible, microelimination in key populations and certain settings remains an invaluable step forward ^24^. Microelimination must be a concerted effort comprising different, complementary initiatives. As highlighted by a *Lancet HIV* editorial ^24^ (p. e605), published before the major disruption caused by the COVID-19 pandemic: “*It is worrying though that a recent survey of BHIVA* [British HIV Association] *members showed that it is becoming more difficult for people to test for HIV, including testing in outreach settings*”.

Projects for expanding rapid testing include various methods for uptake, testing, and engagement that seek to approach the reality of specific groups. Recent articles ^25^ ^,^ ^26^ suggest that casual sex facilitated by dating applications merits discussion in an age of massive social media use and a certain discredit among younger generations regarding the measures adopted in previous diverse settings.

Use of mobile units (trailers) at strategic locations has served as an important alternative for expanding HIV testing since primary care units office hours fail to cover part of the population. Of the four projects analyzed, only *Live Better Knowing* fails to mention this strategy, although it frequently conducts outreach activities in public spaces.

Mobile testing services have attempted to reduce geographic, social, economic, and cultural barriers that prevent individuals from obtaining early HIV/AIDS diagnosis. This strategy is widely used in some countries, and studies ^27^ ^,^ ^28^ ^,^ ^29^ indicate that higher HIV prevalence can be found in individuals tested by mobile units. Projects implemented in Baltimore, United States, and Chiang Mai, Thailand, for example, registered higher testing rates in mobile units than in stationary testing facilities ^28^ ^,^ ^29^. But the model has also been criticized since mobile units tend to lose their intended purpose once “parked” in fixed testing locations and the activities mostly reach MSM and *travestis* that frequent LGBTQIAPN+ nightclubs and bars, thus failing to cover the wider MSM and *travesti* population who do not congregate in these venues. A well-balanced combination of mobile and fixed testing locations seems to be the best available strategy, as argued in a systematic review and meta-analysis by Sharma et al. ^30^.

Peer educators participated in all the projects analyzed (in the *Find Out Young*/*Live Better Knowing Young* project they were called “youth mobilizers”). Trained to raise awareness on rapid HIV testing, they played a key role in increasing testing adherence as individuals recruited by peer educators identify with their peers. Wide acceptance of providing saliva samples to youth mobilizers, as in the *Find Out Young*/*Live Better Knowing Young* project confirms it. The international literature lists a series of successful interventions worldwide where peer-educators were a key asset and have been fully incorporated into standard protocols. Newman et al.’s ^31^ scoping review summarize these relevant findings for a pool of countries on the Mekong Region.

Another important strategy that requires further detailed analysis is the distribution of self-tests to adolescents and youth, as in the *Live Better Knowing Young* project in Recife. Recent article ^32^ indicates that this prevention strategy is well-accepted by adolescents and youth, since the fear of stigmatization in healthcare services is their main reason for avoiding them.

Finally, the innovative strategy of “linkage and linkers” was employed by the *The Time is Now* project to ensure immediate uptake by referral services for positive testing and showed promising results in the successful chain-of-care trajectory.

### Governance and civil society: interoperability and cooperation

This analysis adopted the concept of governance described by Lange et al. ^33^, qualifying interaction at the institutional level (public and/or private governance) aimed at specific objectives. The projects for expanding rapid HIV testing clearly display the interoperability of consolidated public governance, integrating administration, civil society, and the community to ensure the projects’ success. Moreover, international cooperation guarantees a link between national and international initiatives, expanding the concept of global governance. Lange et al. ^33^ broadened the landmark concept of “advocacy collations”, explored by two authors of the present paper, in collaboration with another member of our research group, as originally coined by Paul A. Sabatier and applied to the Brazilian context in a former paper ^34^.

International agencies played different roles depending on the project. Some served as funding agencies (e.g., USAID in the *I Want to Get Tested* project, UNODC in the *Live Better Knowing* project, and PEPFAR in *The Time is Now* project). Others were direct participants (e.g., UNICEF in the *Find Out Young*/*Live Better Knowing Young* project), serving as both supporter and implementer. This raises the issue of project sustainability, since the UNICEF proposal took over these roles for a limited period. Government agencies were expected to assume the responsibility later, but faced challenges in guaranteeing ongoing funding and difficulties in coordinating the state and municipal health departments.

Relations between the three government spheres (federal, state, and municipal) resemble a cooperative governance according to *Brazil’s 1988 Federal Constitution* and Organic Health Law and the transfer of responsibilities from the Federal Government to states and municipalities, thereby promoting their autonomy and accountability ^35^. In the current case, however, the states and municipalities have merely supported projects for expanding HIV testing in key populations, underlining the lack of projects led by states and municipalities themselves.

The analyzed documents show that most projects for expanding testing for key populations in Brazil were conducted by civil society organizations in direct partnership with the Brazilian Ministry of Health. NGO participation in health governance has been a characteristic of decision-making processes on a global scale ^36^. Regarding HIV/AIDS, activists and AIDS NGOs have always taken a clear lead in responsive governance in contrast to the vertical top-down power logic, thus democratizing the policy decision-making process.

Ribeiro et al. ^37^ state that issues involving social participation and government collegiate bodies have countered the hierarchical and vertical patterns in the State apparatus and enhanced government transparency in policy development. The *Live Better Knowing* project is a clear example of AIDS NGO leadership. Its team performed all activities (from offering testing to referring positive cases to specialized services) and were free to develop their own strategies in patient approach, uptake, and counseling, thereby breaking with top-down patterns ^38^.

However, NGOs have faced challenges such as shortages in human resources, delays in fund transfer, and setbacks in outreach activities due to the COVID-19 pandemic, to name a few. Administrative and organizational problems, especially involving activity feedback, are also persistent because many of the states and municipalities contacted failed to provide adequate information on the projects’ results and sustainability.

One final interesting point is the innovation proposed by the *The Time is Now* project. Unlike its counterparts, *The Time is Now* includes scientific institutions as collaborators which explains the number of scientific publications about this project and the consistent result presentation. Such partnerships should be fostered in future projects as this synergy seems key to successful initiatives.

## Final remarks

Governance system in the projects for rapid HIV testing in key Brazilian populations results from networking between Brazilian and international stakeholders and institutions, fostered by cooperation between the three government levels (federal, state, and municipal) and civil society.

The projects contributed to expanding efforts in HIV testing and prevention in specific populations, using various strategies such as mobile units, peer education, and innovative community engagement approaches. Difficulties with coordination, funding sustainability, and the impact of external factors such as the COVID-19 pandemic affected their implementation and results. These findings highlight the need to consider organizational, financial, and contextual factors to ensure the success and sustainability of initiatives for expanding HIV testing in key populations as a key public policy for inclusive healthcare and democratic rule of law.
